# Countrywide multi-serotype outbreak of *Salmonella* Bovismorbificans ST142 and monophasic *Salmonella* Typhimurium ST34 associated with dried pork sausages in France, September to January 2021

**DOI:** 10.2807/1560-7917.ES.2023.28.2.2200123

**Published:** 2023-01-12

**Authors:** Maria Pardos de la Gandara, Nelly Fournet, Laetitia Bonifait, Sophie Lefèvre, Marianne Chemaly, Charlotte Grastilleur, Sabrina Cadel-Six, Patrick Fach, Agnès Pignault, Anne Brisabois, Nathalie Jourdan-Da Silva, François-Xavier Weill

**Affiliations:** 1 Institut Pasteur, Université Paris Cité, Unité des Bactéries pathogènes entériques, Centre National de Référence des *Escherichia coli, Shigella* et *Salmonella*, Paris, France; 2 Santé publique France, Saint Maurice, France; 3 ANSES, Ploufragan-Plouzané-Niort Laboratory, Unit of Hygiene and Quality of Poultry and Pork Products, Ploufragan, France; 4 Mission des Urgences Sanitaires, Direction générale de l’alimentation, Paris, France; 5 ANSES, Laboratory for Food Safety, *Salmonella* and *Listeria* Unit, Maisons-Alfort, France; 6 ANSES, Laboratory for Food Safety, IdentyPath Genomics Platform, Maisons-Alfort, France; 7 ANSES, Strategy and Programs Department, Research and Reference Division, Maisons-Alfort, France

**Keywords:** Salmonella, multi-serotype, outbreak, Bovismorbificans, Typhimurium, cgMLST

## Abstract

The French National Reference Centre for *Escherichia coli, Shigella* and *Salmonella* (FNRC-ESS) detected two human clusters of 33 cases (median age: 10 years; 17 females) infected by *Salmonella enterica* serotype Bovismorbificans, ST142, HC5_243255 (EnteroBase HierCC‑cgMLST scheme) in September–November 2020 and of 11 cases (median age: 11 years; seven males) infected by *S. enterica* serotype 4,12:i:-, ST34, HC5_198125 in October–December 2020. Epidemiological investigations conducted by Santé publique France linked these outbreaks to the consumption of dried pork sausages from the same manufacturer. *S.* Bovismorbificans and *S.* 4,12:i:- were isolated by the National Reference Laboratory from different food samples, but both strains were identified in a single food sample only by qPCR. Three recalls and withdrawals of dried pork products were issued by the French general directorate of food of the French ministry for agriculture and food in November 2020, affecting eight supermarket chains. A notification on the European Rapid Alert System for Food and Feed and a European urgent enquiry on the Epidemic Intelligence Information System for Food and Waterborne Diseases and Zoonoses (EPIS-FWD) were launched. No cases were reported outside France. Outbreaks caused by multiple serotypes of *Salmonella* may go undetected by protocols in standard procedures in microbiology laboratories.

**Key public health message**

**What did you want to address in this study?**

*Salmonella enterica* subsp. *enterica* is the main cause of food-borne diarrhoeal infections in Europe. Present diagnostic protocols target single-agent infections, but not multi-serotype infections. We wanted to describe a recent multiple-serotype *Salmonella* outbreak in humans in France in 2020, which led to an international investigation.

**What have we learnt from this study?**

The outbreak affected 44 people in association with the consumption of dried pork sausages contaminated by two different serotypes of *Salmonella*. The investigations identifying multiple serotypes of *Salmonella* in the food were complicated and required the collaboration of seven different institutions. The identification of the two serotypes was only possible in a single food sample.

**What are the implications of your findings for public health?**

This study highlights the need to improve the procedures to better detect mixed contaminations of food products by different serotypes of *Salmonella*, that we believe may go undetected with the present standard laboratory procedures.

## Background

*Salmonella enterica* is a major cause of gastroenteritis, with 180 million cases globally per year (9% of all infectious gastroenteritis cases) and is responsible for almost half (41%) of the deaths associated to the diarrhoeal disease. *Salmonella* shows the highest rates of demonstrated association to food-borne infection, i.e. 52% for non-typhoidal salmonellosis [1]. In 2019, 87,923 confirmed cases of salmonellosis in humans were reported in Europe, with a European Union (EU) notification rate of 20.0 cases per 100,000 population; *Salmonella* caused 26.6% of all food-borne outbreaks [2]. In France, *Salmonella* remains the main cause of food-borne illness–associated hospitalisation and death [3,4].

Three serotypes are responsible for the majority of *Salmonella* infections in Europe: Enteritidis, Typhimurium and its monophasic variant (1,4,[5],12:i:-), together representing 70.3% of the 79,300 confirmed human cases with a known serotype in 2019. After poultry, pork is the most frequent source for salmonellosis in Europe (31%), and it has become the most frequent source for *Salmonella enterica* serotype Typhimurium and its monophasic variant 1,4,[5],12:i:-. In France, pork is suspected to be responsible for half of the salmonellosis cases reported every year [2,5,6].

*S. enterica* serotype Bovismorbificans is a relatively frequent food-borne pathogen (57 cases/year in France from 2012–20, and it was the 13^th^ most frequently isolated serotype among human-identified *Salmonella* infections in Europe in 2019 [2]. Serotype Bovismorbificans is often identified in association with consumption of contaminated vegetables [7-11]. However, it has also been recently involved in outbreaks linked to horse and pork meat in Australia and France [12,13].

## Outbreak detection

Between 27 October and 6 November 2020, the French National Reference Centre for *E. coli, Shigella* and *Salmonella* (FNRC-ESS), at the Institut Pasteur in Paris, France, detected through the routine sequencing and clustering of *Salmonella* spp. a cluster of 14 human isolates of *S.* Bovismorbificans ST142, with HC5_243255. Cases resided in four different regions in France, with ages ranging from 1 month to 48 years. On 24 December 2020, the FNRC-ESS identified another cluster of nine cases of *S.* 4,12:i:-, ST34, HC5_198125. Patients lived in seven regions in France, with ages ranging from 4 to 42 years old. 

The French National Institute for Public Health (Santé publique France, SpF), initiated the outbreak investigations, in collaboration with the French general directorate of food (DGAL) and the National Reference Laboratory at the French National Agency for Food Security (LNR-ANSES) to identify the source of the outbreak and implement control measures. Epidemiological investigations showed the consumption of the same food product was associated to most of cases.

We present here our investigation of two associated food-borne outbreak clusters of salmonellosis in France, one caused by *S.* Bovismorbificans ST142 and the other caused by *S.* 4,12:i:-, ST34. We also aimed to draw attention to the possibility of multi-serotype *Salmonella* outbreaks occurring and the challenges associated with their identification.

## Methods

### Case definition

A confirmed case was defined as a person with a laboratory-confirmed isolate of *S.* Bovismorbificans, ST142 or *S.* 4,12:i:-, ST34, belonging to the outbreak phylogenetic clusters: HierCC HC5_243255 or HC5_198125, respectively, or at five or less allelic distance (AD) to another isolate in the cluster, by the EnteroBase HierCC scheme on core genome multilocus sequence typing (cgMLST) [14].

All cases were notified by the FNRC-ESS to SpF upon identification; name, date of birth, sex and postal code were included in the notification. There was no timeframe in the case definition.

### Epidemiological investigation

Following the first notifications of the two clusters, and then after each new case detection by the FNRC-ESS, SpF interviewed patients using a standardised *Salmonella* trawling questionnaire, to collect information on dates of symptoms onset, hospitalisation, food consumption, other exposures in the 7 days before illness, places of food purchase and numbers of loyalty cards, if available. A descriptive analysis of the cases was conducted, and relevant information were shared with the DGAL for an investigation of food products. 

No further analytical studies (case control or cohort) were carried out for this outbreak and no follow-up was performed for the identified cases, because the outbreak ceased following the food recalls and withdrawals.

### Human microbiological investigation

#### Clinical sample collection

Bacterial isolates were sent to the FNRC-ESS by laboratories of clinical microbiology. As part of the routine procedure, all laboratories in France are encouraged to send all *Salmonella* isolates for surveillance on a voluntarily basis. In 2020, 7,181 isolates were received and analysed at the FNRC-ESS, of which 80 belonged to serotype Bovismorbificans and 1,820 to the monophasic variant of serotype Typhimurium (4,[5]12:i:-).

All 44 isolates in this outbreak were obtained from stool samples, except for two isolates of the *S*. Bovismorbificans ST142 HC5_243255 cluster that were obtained from blood cultures.

#### Whole genome sequencing

Whole genome sequencing (WGS) was performed as part of routine procedures at the FNRC-ESS. WGS was carried out at the Plateforme de microbiologie mutualisée (P2M) from the Pasteur International Bioresources network (PIBnet, Institut Pasteur, Paris, France). The MagNAPure 96 system (Roche Diagnostics) was used for DNA extraction, libraries were prepared using the Nextera XT kit (Illumina) and sequencing was done with the NextSeq 500 system (Illumina). Serotype prediction was done by in-house scripts based on MLST [15], *fliC* and *fljB* gene databases (FNRC-ESS internal flagellin database, unpublished). 

Genomic sequences from all *S*. Bovismorbificans ST142 and *S*. 4,12:i:- ST34 isolates received at the FNRC-ESS were deposited, per routine, into EnteroBase (https://enterobase.warwick.ac.uk) [16]. Phylogenetic analysis was performed by two different approaches integrated into EnteroBase: single nucleotide polymorphism (SNP) analysis (maximum-likelihood trees) and cgMLST (HierCC and minimum spanning trees based on AD. 

Antimicrobial resistance profiles were inferred using the Resfinder 4.1 tool (https://cge.food.dtu.dk/services/ResFinder) [17].

Classical sero-agglutination was performed on three selected isolates of the ST34 cluster.

### Food product investigation

The epidemiological investigations carried out by SpF pointed towards dried pork sausages produced by Manufacturer X. Trace-back food investigations on those sausages were directed by the DGAL in November 2020. Biological food sampling was performed at different levels: (i) self-controls by Manufacturer X (1 sample per batch and environmental), (ii) reinforced self-controls by Manufacturer X (8 samples from Batch I), (iii) self-controls by Supermarket Chain 1 and (iv) official controls by the local food agri-food laboratories (Laboratoire Départemental d’Analyses (LDA)) (1–5 samples per batch for 12 batches, including Batch I, with no samples available from Batch II).

Additionally, a leftover food sample provided by one patient was sent to the LNR-ANSES for analysis. All analyses followed the NF EN ISO 6579–1 standards [18]. *Salmonella* isolates were serotyped according to the White-Kauffmann-Le Minor scheme [19]. Non-routine in-house quantitative real-time PCR (qPCR) assays targeting *S.* Bovismorbificans and *S.* Typhimurium/*S.* 1,4,[5],12:i:- were performed on the leftover food samples. Briefly, 1 ml of the pre-enrichment broths were DNA extracted using the InstaGene matrix following the recommendation of the manufacturer (Bio-Rad) and 5 µl of the DNA were tested by qPCR in a CFX apparatus (BioRad) (see Supplementary Table S1 for the primer and probe sequences used in this study).

The *Salmonella* food isolates were eventually sequenced by the LNR-ANSES using Illumina technology as described elsewhere [20]. Three tools available at the Center for Genomic Epidemiology (CGE) (http://genomicepidemiology.org) were used to analyse the genomes: SeqSero 1.2, MLST and ResFinder 2.1. These isolates were compared with the human isolates on EnteroBase (both SNP and cgMLST approaches).

## Results

### Epidemiological investigation

**Figure 1:**
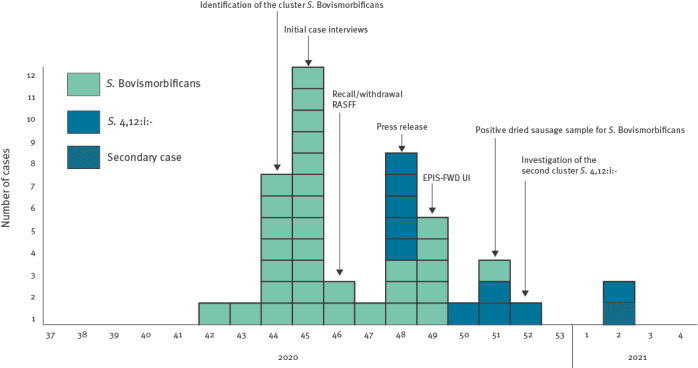
Epidemic curve of *S.* Bovismorbificans ST142 HC5_243255 (n = 33) and *S*. 4,12:i:- ST34 HC5_198125 (n = 11) cases by week of identification at the FNRC-ESS, France, September 2020–January 2021

EPIS-FWD: Epidemic Intelligence Information System or Food and Waterborne Diseases; RASFF: Rapid Alert System for Food and Feed; *S*.: *Salmonella*, UI: urgent enquiry.

To determine the source of the outbreak, epidemiological investigations were conducted during the 3 months following the identification of the first genomic cluster, starting on 6 November 2020, and until the outbreak ended. Of the 33 cases of *S.* Bovismorbificans, 19 were children less than 13 years of age, median age was 10 years (range: 2 months–69 years), and 17 were female. SpF successfully interviewed 23 cases (or their parents); the remaining nine cases were not reachable (no contact information available or not answering the phone call). Symptom onset ranged from 22 September to 10 November 2020 (Figure 1) and seven patients required hospitalisation. Twenty-three cases reported grocery shopping at one specific supermarket chain; 22 cases indicated the consumption of dried pork sausages and 17 allegedly purchased the same Brand A. For 19 cases, the loyalty card number for Supermarket Chain 1 was provided, which confirmed the purchase for 15 cases and helped to identify unreported purchases by four additional cases.

Secondary case refers to a 2-month-old infant who may have acquired the infection through cross contamination from another case in the household.

**Figure 2:**
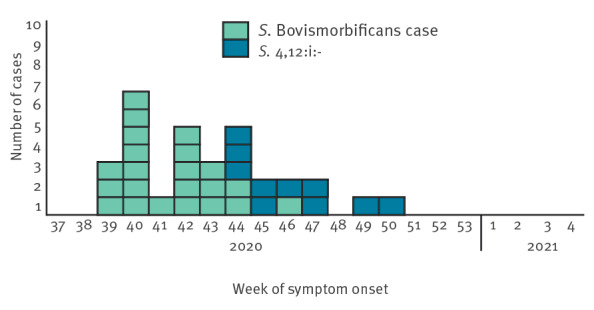
Epidemic curve of *S.* Bovismorbificans ST142 HC5_243255 (n = 23^a^) and *S.* 4,12:i:- ST34 HC5_198125 (n = 10) interviewed cases by week of symptom onset, France, September–December 2020

The second cluster, caused by *S.* 4,12:i:- ST34, had 11 cases with ages ranging from 2 months to 49 years (median: 11 years), seven of whom were male. Ten cases were interviewed; Case 11 was an infant aged 2 months, thus a secondary case. Three cases were hospitalised, and symptom onset ranged from 27 October to 30 November. One case reported a later date of onset, on 12 December (Figure 2). Nine cases reported the recent consumption of dried pork sausages and eight of them indicated Brand A, purchased in Supermarket Chain 1 stores. All nine patients provided the number of their loyalty card, and the purchase was thus confirmed for seven of them. There were no deaths associated to either outbreak.

*S.*: *Salmonella*.

^a^ Date of symptom onset unknown for one case.

### Human microbiological investigation

**Figure 3:**
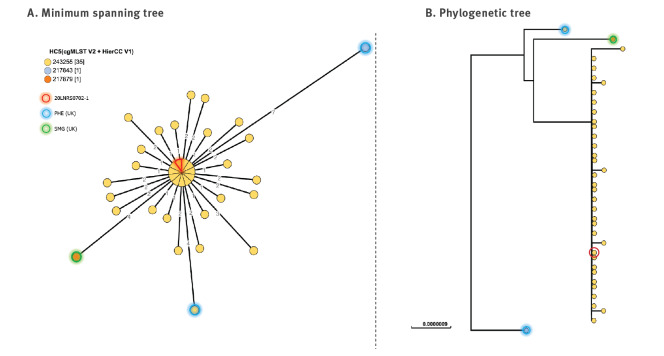
Phylogenetic visualisation of the *S.* Bovismorbificans HC5_243255 cluster (n = 33) in relation to other ST142 genomes (n = 4) up to 17 February 2021, France

AD: allelic distance; cgMLST: core genome multilocus sequence typing; SNP: single nucleotide polymorphism.

On 27 October 2020, the FNRC-ESS notified to SpF a cluster of eight *S.* Bovismorbificans ST142 isolates sharing a new HC5 profile (HC5_243255) (Figure 3), all sampled within a period of 12 days (22 September–5 October). By the end of 2020, a total of 33 isolates were identified, with isolation dates ranging between 22 September and 16 November. The isolates were inferred to be susceptible to all antibiotics.

The trees were constructed on EnteroBase (http://enterobase.warwick.ac.uk), selecting ST142 (n = 37) genomes clustering at less than 10 AD from the central node of the HC5_243255 cluster. Two genomes were uploaded by the United Kingdom (UK) Health Security Agency, appearing on the EnteroBase site as Public Health England (PHE; blue circles) and one genome was uploaded by the Scottish National Health System (NHS, SMG on EnteroBase), from the UK (green circle). The isolate from the food sample is 20LNRS0702–1 (red circle).

A. Minimum spanning tree on cgMLST by the EnteroBase ‘MSTree V2’ algorithm. Dots in the tree represent genomes. When genomes are at 0 AD, they form a bigger dot, where each genome is a sector in the dot. All HC5_243255 genomes are coloured in yellow, and that includes the 33 outbreak genomes – all at 0 to 3 alleles from one another –  n = 1 genome from the food sample (outlined in red, in the centre of the main node), n = 1 genome from the UK (at 4 AD, circled in blue).

B. Phylogenetic tree constructed with the ‘create SNP project’ tool in EnteroBase, based on the 196 non-repetitive SNPs present in 95% or more of all the queried genomes. Each dot in the tree represents one genome. Colours correspond to different HC5, and HC5_243255 (to which the outbreak cluster belongs) is here represented in yellow.

**Figure 4:**
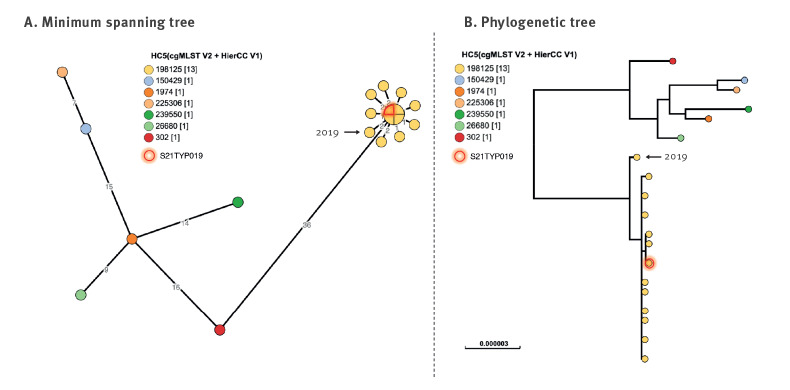
Phylogenetic visualisation of the *S*. 4,12:i:- ST34 HC5_198125 cluster (n = 11) in relation to other ST34 HC50_2 genomes (n = 7) up to 18 March 2021, France

AD: allelic distance; cgMLST: core genome multilocus sequence typing; SNP: single nucleotide polymorphism.

Between 24 December 2020 and 8 January 2021, the FNRC-ESS identified another cluster of 11 cases of *S*. 4,12:i:- ST34 HC5_198125 (isolation dates ranging from 31 October–14 December) (Figure 4). They were genomically assigned to *S.* Typhimurium because of the presence of both *fliC*:i and *fljB*:1,2 flagellin genes in all the isolates. However, classical sero-agglutination performed on three selected isolates revealed that they were monophasic (i.e. *fljB*:1,2 was not expressed). Further analyses revealed that this was due to an insertion sequence that prevented fragment H (including *hin* and the *fljB* promoter) from inverting its genetic configuration and therefore, expressing *fljB*. The outbreak genetic configuration of invertible Fragment H scheme is shown in Supplementary Figure S1.

The trees were constructed on EnteroBase (http://enterobase.warwick.ac.uk), selecting ST34 genomes (n = 19) clustering at less than 10 AD from the node of the HC5_198125 cluster. The isolate from food origin is S21TYP019 (circled in red).

A. Minimum spanning tree on cgMLST by the EnteroBase ‘MSTree V2’ algorithm. Dots in the tree represent genomes. When genomes are at 0 AD; they form a bigger dot, where each genome is a sector in the dot. All cluster members (yellow circles) are at 0 to 2 alleles from one another, which corresponds to HC5_198125. One genome clustering together with the outbreak genomes dated from 2019 (arrows) and was therefore not considered as part of the outbreak.

B. Phylogenetic tree constructed with the ‘create SNP project’ tool in EnteroBase, based on the 125 non-repetitive SNPs present in 95% or more of all the queried genomes. Each dot in the tree represents one genome. Colours correspond to different HC5, and the outbreak cluster (HC5_198125) is here represented as yellow. The 2019 isolate was well separated from the 2020 outbreak cluster, while the food genome S21TYP019 clustered with the genomes of human origin in 2020.

### Food product trace-back investigation 

Trace-back investigations on Batch I (suspected batch for cases belonging to the *S.* Bovismorbificans outbreak cluster) performed in November 2020 by the manufacturer (8 samples) and by the LDA (3 samples) were negative for *Salmonella* spp.

However, dried pork sausage leftover samples belonging to Batch I provided by one of the cases were sent to the LNR-ANSES. *S.* Bovismorbificans was isolated and identified by the standard methods, including sero-agglutination, and detected directly in the food sample by an in-house qPCR targeting serotype Bovismorbificans (Ct values: ca 20). Supplementary Figure S2 shows the qPCR analysis of the food sample. 

In January 2021, following the identification of the second human outbreak cluster caused by *S.* 4,12:i:-, the epidemiological and food investigations uncovered a new batch of incriminated products (Batch II) from the same manufacturer. Reinforced self-control analyses were then performed by Manufacturer X (n = 5 samples) and *S.* 4,12:i:- was isolated from one sample and identified by classical sero-agglutination. No other *Salmonella* serotypes were found. 

A new qPCR assay targeting serotype Typhimurium/1,4,[5],12:i:- was then performed on the remaining DNA extracts from the leftover samples from Batch I that had been previously positive to *S*. Bovismorbificans, and the assay identified *S.* Typhimurium/1,4,[5],12:i:- (Ct value: ca 30) (Supplementary Figure S2). However, by this time, there were no samples available for culture to determine whether *S*. 4,12:i:- ST34 HC5_198125 could be also isolated in the same samples from Batch I in which *S.* Bovismorbificans was previously isolated.

Supplementary Figure S2 shows the qPCR analysis of the food sample, where four genomic targets were aimed: A. *S*. *enterica*, the species to which both outbreak serotypes belong, B. serotype Bovismorbificans and C. and D. two different targets for serotype Typhimurium/4,[5],12:i:-. While qPCR for *S. enterica* and serotype Bovismorbificans had Ct values of ca 20 cycles, the qPCR for *S.* Typhimurium/1,4,[5],12:i:- had Ct values of ca 30 cycles, which means that there was less amount of *S.* Typhimurium/1,4,[5],12:i:-DNA than of *S.* Bovismorbificans in the sample. 

WGS allowed the confirmation of the identity between human and food *Salmonella* isolates. The isolate first obtained from the patient’s leftover samples (Batch I) was *S.* Bovismorbificans, ST142, HC5_243255, clustering together with the human isolates (Figure 3). The second food isolate, obtained during the Manufacturer X’s analyses in January (Batch II), was characterised as *S.* 4,12:i:- ST34 and HC5_198125, clustering together with the second epidemic outbreak isolates detected in humans (Figure 4).

### Outbreak control measures

A first recall and withdrawal were issued on 13 November 2020 by Supermarket Chain 1, 2 weeks after the initial alert by the FNRC-ESS, and 1 week after the first SpF questionnaires [21]. The recall implied two types of Brand A dried pork sausages. On 16 November 2020, as a complement and preventive measure, two additional batches of sausages from Brand B and Brand C produced with the same raw pork meat as Brand A were recalled and withdrawn. They were sold by Supermarket Chains 2 and 3. A third recall and withdrawal were issued in November 2020 for sandwiches of Brand D made with sausages from Manufacturer X, and sold in Supermarket Chains 4, 5, 6, 7 and 8. All suspected batches of dried pork sausages from Manufacturer X with production dates up to 8 December 2020 were removed from the market.

A Rapid Alert System for Food and Feed [22] (RASFF: 2020.5038) alert was posted by the French authorities on 16 November 2020, at the same time as the second recall and withdrawal were issued in France. The contaminated dried pork sausages had been distributed in other five countries: Belgium, Luxemburg, Poland, Portugal and Slovenia. 

On 26 November 2020, the French Ministry for agriculture and food delivered a press release reporting these recalls and withdrawals.

An urgent inquiry (UI-688, later renamed as 2020-FWD-00065) on the Epidemic Intelligence Information System for Food and Waterborne Diseases and Zoonoses (EPIS-FWD, now called EpiPulse) platform operated by the European Centre for Disease Prevention and Control (ECDC) was issued by SpF on 1 December 2020 concerning the *S.* Bovismorbificans outbreak. Germany, Ireland, Norway, and the UK responded with one case each, at 4 to 5 AD from the representative genome provided by the FNRC-ESS. SNP analysis and epidemiological data excluded them from being part of the cluster.

No additional recalls and withdrawals were initiated following the investigations of the second cluster in end of December 2020 and January 2021, because the products were no longer on the market. No related cases were identified outside of France afterwards. Three *S.* Bovismorbificans genomes genetically close to the epidemiological outbreak were identified in EnteroBase from the UK, but no epidemiological link was established with the French outbreak. The last bacterial isolate of human origin for the *S.* Bovismorbificans cluster dates to 16 November, the day of the second and largest product withdrawal and recall. The last bacterial isolate of human origin for the *S*. 4,12:i:- cluster dates to 14 December. Given the lack of new cases in January 2021, the investigations were closed by the end of the month.

As no other manufacturing plants supplied ingredients from common origin were affected, we speculate with a local contamination at Manufacturer X. However, no investigations upstream were performed, as they are not required, and the staff resources were scarce at that time.

## Discussion

We describe a nationwide outbreak of salmonellosis involving 44 cases in two microbiological clusters by two serotypes: 33 cases with *S.* Bovismorbificans ST142 and 11 cases with the monophasic variant of *S*. Typhimurium ST34. Both clusters were associated with the consumption of dried pork sausages produced by the same manufacturer between September and November 2020. Epidemiological investigations pointed to the consumption of Brand A sausages marketed by Supermarket Chain 1. Moreover, the investigation of the supermarket loyalty cards reinforced this association by recording the purchase of the suspected product a few days before the date of symptom onset in most records. 

Control measures were implemented following the epidemiological investigations of the *S.* Bovismorbificans cluster with three recalls/withdrawals in November 2020. The *S.* 4,12:i:- cluster was identified in late December, but symptom onset of most cases occurred by 30 November (except for one case in December). Following the recalls and withdrawals of the suspected products, detection in human cases declined rapidly. International health agencies were informed of the first outbreak cluster (*S.* Bovismorbificans) through RASFF and EpiPulse messages because of the exportation of the product in other European countries. No cases belonging to the first cluster were reported outside France, and no genomes belonging to the second cluster were identified on EnteroBase for any other country.

*S.* Bovismorbificans was only isolated from a food sample provided by a patient. Although post-purchase contamination by the patient might be possible, the epidemiological data and the absence of new cases following the recalls and withdrawals strongly supported the hypothesis that the product was contaminated before the sale.

*S*. Bovismorbificans is not an unusual serotype infecting humans in France, with a mean of 57 cases per year (median: 51 cases) for the past 10 years. The outbreak here presented reflects an increase of 40% over the average in 2020. This serotype has been often involved in human outbreaks in recent years, mostly associated to the consumption of vegetables like alfalfa sprouts, salads, hummus or tahini in Australia, Finland, Switzerland, Germany and the United States [7-11]. However, the association of *S.* Bovismorbificans with pork products has been described previously, in the Netherlands in 2017 [13]. In that outbreak, trace-back investigations led to a Belgian meat processor. Two more outbreaks caused by *S.* Bovismorbificans have occurred in France in 2019 and 2020: an outbreak of 11 cases in which the source could not be identified, at nine AD from the present outbreak (data not shown), and a cluster of 14 cases associated to the consumption of uncooked horse meat, at more than 50 AD from the present cluster [12]. The ANSES *Salmonella* network, as part of the French Laboratory for Food Safety, has collected 147 *S*. Bovismorbificans isolates in France since 2010, principally obtained from pork meat (n = 32 strains), turkey (n = 23 strains), chicken (n = 16 strains) and horse meat (n = 6) (https://www.ANSES.fr/fr/content/inventaire-des-salmonella-dorigine-non-humaine). *S.* Bovismorbificans should be considered as an important hazard in meat, and measures should be reinforced to control its presence, as is done with other serotypes more frequently identified in association with meat consumption.

Outbreaks of salmonellosis in humans caused by *S.* 1,4,[5],12:i:- ST34 are frequent (over 6,400 cases in 2018 in Europe) and in France they are often linked to the consumption of contaminated raw dried pork meat (39.6%) [2]. The Commission Regulation (EC) No 218/2014 provides information on the mandatory monitoring of *Salmonella* in pork at the production chain [23]. Thereby, *S.* Typhimurium and its monophasic variant 1,4,[5],12:i:- are found as the third and second most frequent serotype of *Salmonella* isolated from the swine sector (animals and food), respectively [24]. However, the isolation of the pathogen in food is not always possible, presumably because of the meat curing (salting and drying) process. Our findings suggest that perhaps microbiological standard procedures should be enhanced by implementing checks at earlier steps of the food processing [25,26].

Outbreaks caused by food simultaneously contaminated by several serotypes of *Salmonella* have been reported, but mostly linked to vegetables. In 2008, Finland reported a nationwide outbreak with up to 106 patients infected by either serotypes Newport, Reading or both, in association to the consumption of contaminated iceberg lettuce [27]. In 2009, an outbreak of nine cases infected by serotypes Schwarzengrund and Typhimurium, was linked to a contaminated potato salad served at a reception in the United States [28]. In 2012, a contaminated tahini paste imported from Turkey caused 27 cases of salmonellosis in New Zealand, with three serotypes of *Salmonella* involved: Montevideo, Maastricht and Mbandaka [29]. A large international outbreak with 94 cases infected in the United States and Canada between 2013 and 2014, implied four different serotypes of *Salmonella* (Newport, Hartford, Oranienburg and Saintpaul) contaminating sprouted chia seed powder distributed by several brands in both countries [30]. More recently, in 2019, an international outbreak affecting more than 100 cases in five countries was associated to the consumption of sesame-based products imported from Syria, contaminated by six different serotypes of *Salmonella* [31]. 

In this outbreak, although it was not possible to isolate both *Salmonella* serotypes from one single food or human sample, a qPCR identified both serotypes in the same food sample. This suggested that the food products could have been contaminated at different rates by both serotypes, simultaneously, escaping the standard procedures of microbiology laboratories for the identification of multi-strain contaminations. At this end, the detection of a positive *Salmonella* signal by qPCR in a pre-enrichment sample may be a powerful approach for fast detection and investigation [32-34]. Nevertheless, the method would need to be standardised and a confirmation should always be performed by strain isolation, identification, and genomic characterisation.

Our study has some limitations. The standard laboratory methods for identification of *Salmonella* in clinical microbiology laboratories do not include the testing of several colonies per sample [28], and that could explain the low numbers of multiple serotype infections reported in the literature. However, it seems logical to expect that processed food may be contaminated by a mixture of *Salmonella* strains more frequently than it is observed. In the outbreak presented here, two different serotypes of *Salmonella* were obtained from two different batches of dried pork sausages produced by the same manufacturer during a short period of time. Date of symptom onset and date of purchase of the product in the two clusters overlapped, and both serotypes were detected in the sausage leftovers kept by one case. Unfortunately, it was not possible to isolate the two serotypes from any human or food samples, which is a clear limitation in our study. Similarly, nine cases could not be reached for interviews during the epidemiological investigation, although the 24 successful interviews gave sufficient evidence to initiate the trace-back food investigations.

## Conclusions

We present here two concurrent *Salmonella* *enterica* outbreaks linked to the consumption of dried pork sausages produced during a short period of time by the same manufacturer: the former caused by *S.* Bovismorbificans and the latter by the monophasic variant of *S.* Typhimurium, 4,12:i:-. However, molecular methods revealed a dual contamination of a single food sample analysed. Multi-strain outbreaks may go undetected by the current bacteriological detection approaches. We believe that standard laboratory protocols, both at the clinical and the food control levels, should include procedures to enhance the detection of mixed *Salmonella* serotypes contaminations. Moreover, our report highlights the utility of serotype-specific qPCR assays for investigating food-borne outbreaks.

## Supplementary Data

747000 Supplement
