# Author’s correction for Euro Surveill. 2023;28(2)

**DOI:** 10.2807/1560-7917.ES.2023.28.3.230119c

**Published:** 2023-01-19

**Authors:** Eurosurveillance editorial team

**Affiliations:** 1 European Centre for Disease Prevention and Control (ECDC), Stockholm, Sweden

In the article ‘Countrywide multi-serotype outbreak of Salmonella Bovismorbificans ST142 and monophasic Salmonella Typhimurium ST34 associated with dried pork sausages in France, September 2020 to January 2021’ by Pardos de la Gandara et al., upon publication on 12 January 2023, the title was missing the year of 2020 after September. This was corrected on 13 January 2023 at the request of the authors.

