# Supplementary material for: Countrywide multi-serotype outbreak of Salmonella Bovismorbificans ST142 and monophasic Salmonella Typhimurium ST34 associated with dried pork sausages in France, September to January 2021
Source: Euro Surveill. 2023 Jan 12;28(2):2200123. doi: 10.2807/1560-7917.ES.2023.28.2.2200123 (PMC9837855; doi:10.2807/1560-7917.ES.2023.28.2.2200123)
Supplement: Supplement [file 22-00123_PARDOS_SUPPLEMENT.pdf]

This supplementary material is hosted by Eurosurveillance as supporting information alongside the article « Countrywide multi-serotype outbreak of *Salmonella* Bovismorbificans ST142 and monophasic Salmonella Typhimurium 4,12:i:- ST34 associated with dried pork sausages in France, September to January 2021 » on behalf of the authors who remain responsible for the accuracy and appropriateness of the content. The same standards for ethics, copyright, attributions and permissions as for the article apply. Supplements are not edited by Eurosurveillance and the journal is not responsible for the maintenance of any links or email addresses provided therein.

Supplementary Table S1. Primers and probe sequences used in this study.

Primer or probe	Sequence 5' -> 3'	Target gene	Target taxa	Reference
invA_176_F	CAACGTTTCCTGCGGTACTGT	*inv*A gene	*S. enterica*	[30]
invA_291_R	CCCGAACGTGGCGATAATT			
Probe: SCS-Salmo2	CTCTTTCGTCTGGCATTATCGATCAGTACCA			
PF-179	CCGTATACGGCTCGCACG	CRISPR2	*S.* Bovismorbificans	This study
PF-181	ACTCAGTTGGTTTGCGCACG			
Probe: Taq-91	AATACCGGGCAGGAAATGAATGAGACGC			
STM2-F	AGATATTCCGTAGCAATTGAGTTG	*pgl*X gene	*S.* Typhimurium or its monophasic variant 4,[5],12:i:-	[31]
STM2-R	AATAGCTAAAAATGACTGGGACTC			
STM2-P	TGTGTTCAAGCAATGGTGAACAAACATAATCCC			
fliA-IS200F	CATTACACCTTCAGCGGTAT	*fli*A gene	*S.* Typhimurium or its monophasic variant 4,[5],12:i:-	[32]
fliA-IS200R	CTGGTAAGAGAGCCTTATAGG			
Probe: fliA-IS200-probe2	CGGCATGATTATCCGTTTCTACAGAGG			

The primers and hydrolysis probes targeting *S.* Bovismorbificans were designed by hand on chromosome of *S.* Bovismorbificans strain 02-7038 CRISPR2 repeat region (JF725071.1) and validated using 900 bacterial strains and 10 *Salmonella* surrogate plasmids. Strains used for validation were divided into *S. enterica* subsp. *enterica* strains (n=798) corresponding to 64 different serovars, and *S. enterica* subsp. other than *enterica* (n=38) and strains other than *Salmonella* (n=64). The exclusivity and inclusivity of the three PCR assays was 100% (data not shown).

Supplementary Figure S1. Outbreak genetic configuration of invertible Fragment H scheme.

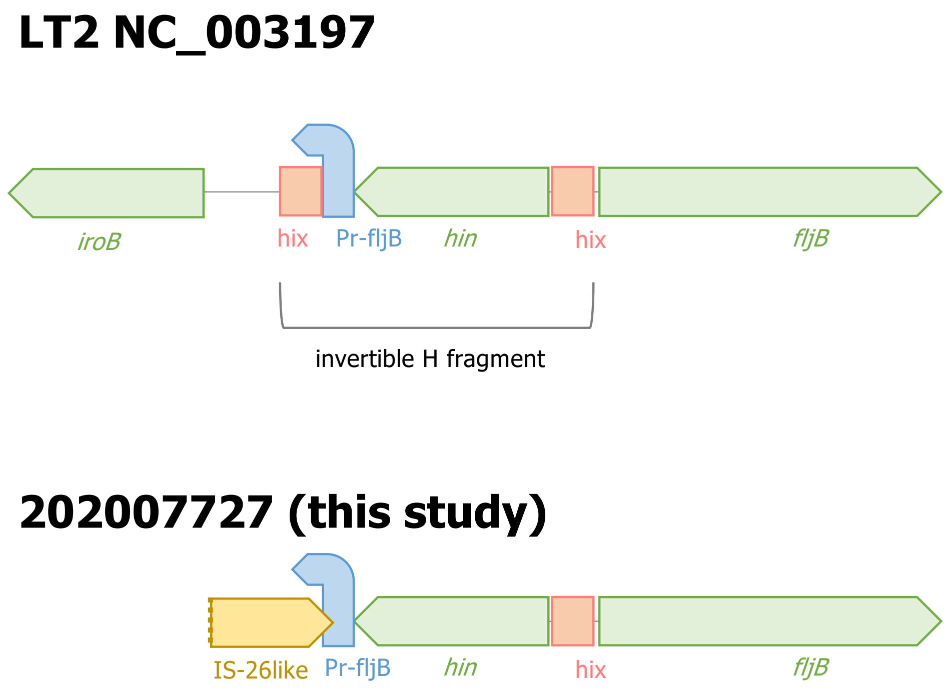

The classical structure of the invertible fragment H in *S.* Typhimurium contains *hin* DNA-invertase and the promoter of fljB, flanked by two *hix* sequences. In the isolates belonging to the ST34 HC5_198125 second outbreak cluster in this study, an insertion sequence of the IS-26 family was inserted upstream to the promoter of *fljB* in the invertible H fragment, preventing fragment H (including *hin* and the *fljB* promoter) from inverting its genetic configuration. This blocked permanently the expression of *fljB*. This is why these isolates were considered as monophasic, in spite of the presence of both *fliC*:i and *fljB*:1,2 flagellin genes in their genomes.

Supplementary Figure S2. Data output from qPCR analysis of the DNA extracts from the pre-enrichment broths (batch I).

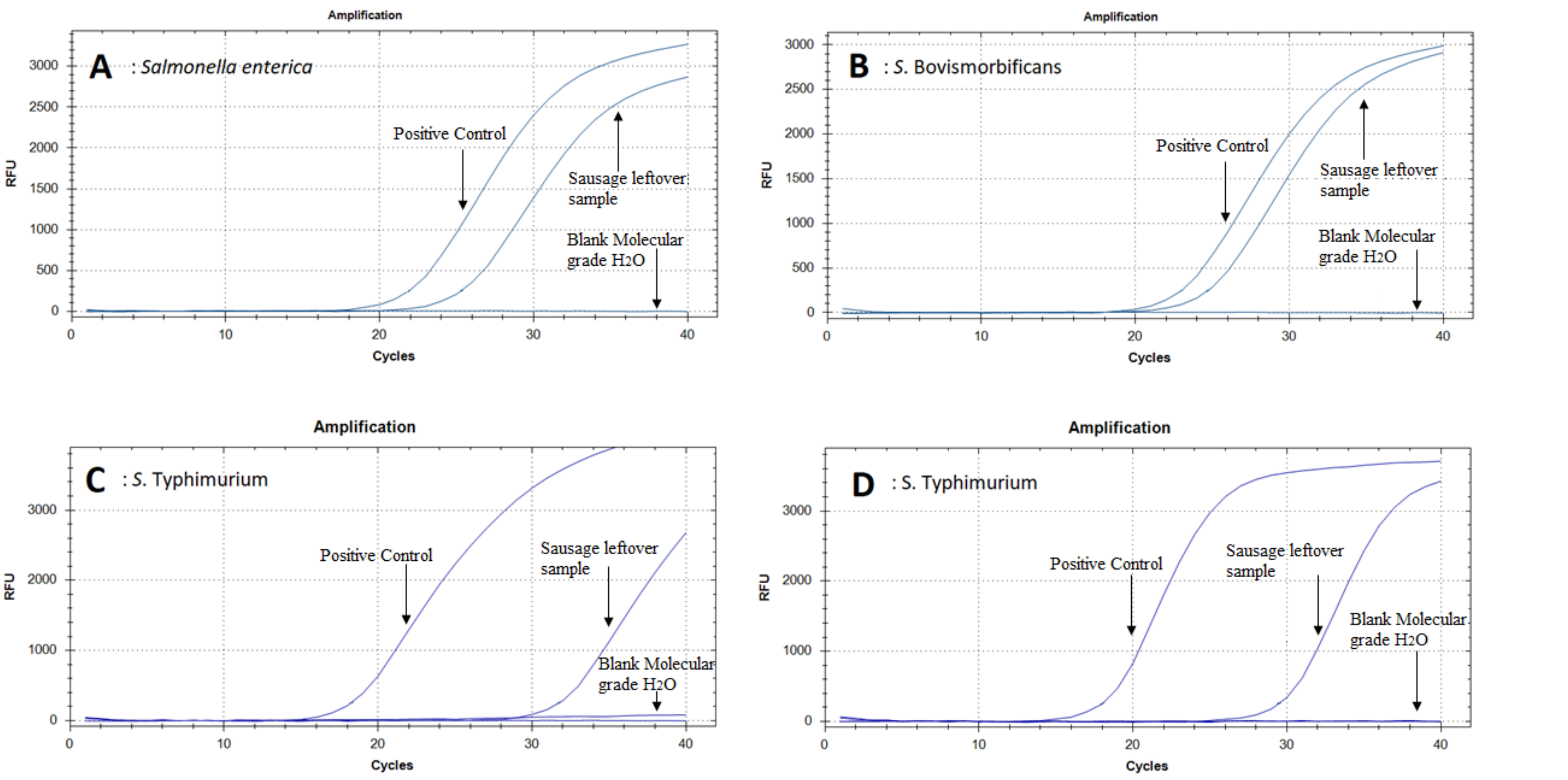

Non-routine in-house quantitative real-time PCR assays (CFX apparatus, BioRad)) targeting S. Bovismorbificans and S. Typhimurium/S. 1,4,[5],12:i:- performed in the leftover food sample from Batch I ('Sausage leftover sample') A: curve of the qPCR targeting *S. enterica,* showing Ct values of ca. 20 cycles for the food sample. B: curve of the qPCR results targeting *S.* Bovismorbificans, showing Ct values of ca. 20 cycles for the food sample. C: curve of the qPCR targeting *S.* Typhimurium/monophasic variant*,* showing Ct values of ca. 30 cycles for the food sample. D: curve of the qPCR targeting *S. enterica,* showing Ct values of ca. 20 cycles for the food sample. Primers and probes are detailed in Supplementary Table S1.
